# Synthetic microbial communities for plant growth promotion and soil-borne disease suppression: design principles, mechanisms, and translational challenges

**DOI:** 10.3389/fmicb.2026.1942488

**Published:** 2026-09-18

**Authors:** Peiwen Cai, Fengjuan Zhao, Ziguo Zhao, Xiancui Zhang, Choufei Wu

**Affiliations:** 1 School of Life Sciences, Huzhou Normal University, Huzhou, China; 2 College of Biological and Pharmaceutical Engineering, Shandong University of Aeronautics, Binzhou, China

**Keywords:** biological control, plant growth promotion, rational design, rhizosphere microbiome, synthetic microbial communities

## Abstract

Declining soil health and soil-borne diseases threaten sustainable crop production, while single-strain inoculants often perform inconsistently in the field due to poor establishment, limited persistence, and narrow functional capacity within resident microbiomes. Synthetic microbial communities (SynComs), assembled from defined and functionally characterized microorganisms, offer a tractable strategy to improve plant growth and suppress disease through complementary and redundant functions. This review critically examines recent advances in plant-associated SynComs, emphasizing rational design over empirical strain mixing. We compare construction strategies based on core microbiome mining, trait-based screening, microbial interaction networks, host and niche adaptation, and multi-omics- or model-guided assembly. Available available evidence suggests that no single SynCom construction strategy is universally superior; rather, robust design requires an integrated, context-specific approach combining host- or core-microbiome-guided candidate selection, trait validation, interaction-informed assembly, and model-guided optimization. We synthesize direct and plant-mediated mechanisms, including nutrient mobilization, phytohormone modulation, stress alleviation, niche pre-emption, resource competition, antibiosis, rhizosphere restructuring, and immune priming. Evidence from representative crop-pathogen systems is evaluated based on mechanistic support, community stability, and validation under non-sterile greenhouse and field conditions. Major barriers to deployment include context-dependent efficacy, instability of community composition and function, formulation and delivery constraints, quality control, ecological safety, and regulatory uncertainty. We propose a design-to-deployment framework linking community assembly with ecological validation, formulation engineering, and multi-environment testing, providing a plant-centered roadmap for developing reliable SynCom-based biofertilizers as well as biocontrol products.

## 1 Introduction

Plants are colonized by highly diverse and functionally specialized microbial communities across different tissues, collectively known as the plant microbiota. In particular, microorganisms inhabiting the rhizosphere are key determinants of plant development, health, and fitness in agroecosystems, functioning as essential partners within the plant holobiont and reflecting long-standing host–microbe coevolution (Philippot et al., 2013; Vandenkoornhuyse et al., 2015; Andreote and Pereira, 2017; Pantigoso et al., 2022). These microbial assemblages expand the genetic and metabolic potential of their host and are frequently described as the plant’s “second genome” (Berendsen et al., 2012; Li X. et al., 2024). Although bacteria and fungi are the dominant and most extensively characterized components of plant-associated microbiomes, increasing evidence indicates that archaea, viruses, nematodes, and protists also play important roles in shaping microbiome assembly and promoting plant performance (Gao et al., 2019; Dolja et al., 2020; Trivedi et al., 2020). Because the present review focuses on SynComs, we primarily consider bacterial and fungal members while acknowledging that other microbiome-associated biota can influence plant microbiome assembly and function. These studies indicate that plant-associated microbiome functions are distributed across multiple taxa and are strongly conditioned by host genotype and environmental context. This functional and ecological context dependence provides the rationale for developing defined communities whose composition, interactions, and performance can be experimentally evaluated and rationally optimized.

Among the bacterial and fungal taxa that dominate this community, however, a subset has evolved as pathogenic colonizers of root tissues, disrupting the otherwise beneficial host–microbiome relationship and giving rise to one of the most persistent threats in agroecosystems: soil-borne disease. Soil-borne pathogens such as *Fusarium oxysporum*, *Alternaria solani*, and *Ralstonia solanacearum* invade plant root systems and vascular tissues, causing devastating soil-borne diseases (Quan et al., 2026). These diseases are characterized by extensive spread, long-term pathogen persistence in soil, and notorious difficulty in control, making them a major bottleneck constraining the sustainable development of global agriculture. This problem is particularly pronounced in intensive cropping systems, where frequent monoculture disrupts soil microecological balance and further exacerbates disease incidence (Mao and Jiang, 2021; Zeeshan Ul Haq et al., 2023).

For decades, agricultural production has relied primarily on chemical pesticides and disease-resistant cultivars to suppress soil-borne diseases. However, the overuse of chemical agents readily induces soil contamination, the emergence of pathogen resistance, and residual toxic substances in agricultural products, raising serious concerns for ecological and food safety (Ahmad et al., 2018). Meanwhile, conventional resistance breeding is constrained by the limited diversity of resistance gene sources, the ease with which such resistance is overcome by evolving pathogens, and lengthy breeding cycles, rendering it inadequate for addressing the diverse disease-control demands of crops under complex field conditions (Wulff and Moscou, 2014; Singh et al., 2015). Consequently, the targeted utilization of the plant microbiota is gaining increasing attention as a sustainable avenue for improving food production systems. Such strategies are especially important as modern agriculture faces mounting pressures from climate change, soil deterioration, reduced reliance on synthetic pesticides, and the need to sustain profitable crop yields (Figueiredo and Lopes, 2021; Raaijmakers and Kiers, 2022; Hanif et al., 2024; Compant et al., 2025).

Microbial inoculants have attracted considerable attention as biological tools for plant growth promotion and disease suppression. Current research has largely focused on individual strains, such as *Bacillus subtilis* (Pérez-Lorente et al., 2025; Ullah et al., 2025) and *Pseudomonas fluorescens* (Riera et al., 2023; Yang et al., 2023; Habibi et al., 2024), with efforts centered on screening superior strains exhibiting plant growth-promoting potential or antagonistic activity to formulate biocontrol products targeting specific pathogens. However, single-strain inoculants often show inconsistent performance in the field. Their efficacy may be restricted by limited functional spectra, poor environmental adaptation, weak persistence in the rhizosphere, and competition with indigenous microbiota (Yin J. et al., 2022). Moreover, single-strain usually depend on one or a few mechanisms, such as antimicrobial metabolite production or niche occupation, and may be insufficient for controlling complex disease systems involving multiple pathogens or fluctuating soil conditions (Chater et al., 2010). For example, impaired root colonization of *B. subtilis* under sucrose-deficient conditions can reduce its biocontrol efficiency against soil-borne diseases (Tian et al., 2021). These constraints have substantially hindered the industrialization and large-scale application of single-strain inoculants. The central challenge is not simply to identify individual beneficial microorganisms, but to determine which combinations can establish, coexist, and maintain complementary functions within resident soil microbiomes.

Synthetic microbial communities (SynComs) offer a robust framework for overcoming some of these limitations. SynComs are defined microbial consortia assembled from selected isolates with known taxonomic identities, functional traits, and ecological interactions (van Leeuwen et al., 2023). Importantly, single-strain inoculants can provide effective plant growth promotion or disease suppression in well-matched systems, and SynComs should therefore not be regarded as intrinsically superior to single-strain inoculants. Rather, comparative studies suggest that defined consortia may improve colonization, functional stability, or disease suppression when their members are ecologically compatible, functionally complementary, and adapted to the target host and soil environment. These potential advantages remain system-dependent, and their persistence across locations, seasons, and agricultural management regimes requires further validation. Accordingly, the key question is not whether greater community complexity is automatically beneficial, but which combinations of strains generate reproducible functions without compromising compatibility or ecological establishment. Recent studies have demonstrated the potential of SynComs in major crop systems, including tomato, rice, wheat, and other economically important crops (Panchal et al., 2025). For example, a cross-kingdom synthetic community has been shown to activate genes within the salicylic acid (SA) and jasmonic acid (JA) signaling pathways and enhance the expression of active enzymes such as chitinase, effectively suppressing *F. oxysporum* f. sp. *lycopersici* (FOL) in tomato–providing further evidence for the green control of soil-borne diseases (Zhou et al., 2022).

Here, we review recent advances in plant-associated SynComs for plant growth promotion and soil-borne disease suppression. This article is a critical narrative review rather than a formal systematic review or meta-analysis. We first summarize major design principles and construction strategies, including core microbiome-guided selection, trait-based screening, interaction-informed assembly, host- and niche-adapted design, and multi-omics- or model-guided rational design. We then synthesize the mechanisms by which SynComs promote plant growth and suppress soil-borne diseases, with particular emphasis on nutrient mobilization, growth-regulating metabolites, niche competition, antimicrobial activity, immune priming, and resident microbiome remodeling. Finally, we discuss representative crop–pathogen applications and highlight translational challenges related to field stability, formulation, delivery, quality control, biosafety, and regulatory assessment. The representative application studies were selected to illustrate major SynCom design strategies, mechanisms, crop–pathogen systems, and translational constraints. Because these studies used heterogeneous experimental settings, comparators, and outcome measures-including disease incidence, severity indices, pathogen density, inhibition rate, biomass, and yield-we report quantitative outcomes at the individual-study level rather than calculating a pooled effect size. By linking ecological design principles, mechanistic understanding, application evidence, and deployment barriers, this review aims to provide a conceptual framework for advancing SynComs from experimental microbiome tools to reliable agricultural technologies.

## 2 Design principles for plant-associated SynComs

Synthetic microbial communities (SynComs) design should balance five competing goals: colonization, complementarity, compatibility, controllability, and commercial feasibility (Mehlferber et al., 2024). Throughout this review, the five Cs are used as cross-cutting evaluation criteria rather than as five independent construction methods. Colonization refers to the establishment and persistence of SynCom members in the target host-associated niche; complementarity refers to functional division of labor and strategically useful redundancy; compatibility refers to stable coexistence without detrimental intra-community interactions; controllability refers to defined membership, reproducibility, traceability, and manageable complexity; and commercial feasibility encompasses manufacturability, formulation stability, shelf life, cost, and regulatory tractability. The five construction strategies described below differ in the extent to which they address these criteria, and no single strategy is expected to optimize all five dimensions independently (Northen et al., 2024). SynComs are simplified and reproducible microbial consortia assembled from defined microbial isolates to mimic key functions of natural microbiomes or to generate desired functions under controlled ecological contexts. Recently, the term “defined microbial communities” has been proposed as an alternative to “synthetic microbial communities” for practical applications, emphasizing the use of cultured, taxonomically characterized, and deliberately assembled microbial consortia (Koch et al., 2026).

In plant systems, SynCom members are usually derived from host-associated or soil microbiomes and selected according to their potential roles in root colonization, nutrient mobilization, stress tolerance, pathogen suppression, or microbiome remodeling. In some cases, engineered strains may also be incorporated to introduce or enhance specific functions. The key distinction between SynComs and conventional mixed inoculants lies in their defined membership and design logic: each member is included on the basis of a hypothesized ecological or functional contribution rather than by empirical mixing alone (Li C. et al., 2024). For the purposes of this review, we use the term SynCom primarily for reproducible communities of cultured microorganisms with defined membership that are deliberately assembled according to explicit ecological or functional criteria. However, because terminology remains inconsistent across the plant microbiome literature, we also discuss a limited number of defined co-inoculation or microbial-consortium studies when they provide mechanistic or translational evidence directly relevant to SynCom design. Such cases are identified explicitly as co-inoculations, defined consortia, or SynCom-based formulations rather than treated as equivalent to rationally designed SynComs. In contrast to the high complexity and stochasticity of natural microbial communities, SynComs are assembled according to core principles of functional complementarity, metabolic synergy, and ecological compatibility, whereby strains with favorable compatibility are selected and combined to achieve metabolic division of labor, signaling interactions, and coordinated resource utilization among community members. Such design enables stable and predictable biological functions within specific habitats, making SynComs a central research model for simplifying natural microbiome structures and dissecting the underlying mechanisms of community function (Song et al., 2026).

Accordingly, the construction of SynComs has evolved from early strain combinations based largely on empirical screening toward a rational design process informed by core microbiome composition, functional traits, strain-strain interactions, key taxa within co-occurrence networks, and multi-omics-based computational prediction (Jing et al., 2024; Yu et al., 2025). As discussed below, each strategy emphasizes a different subset of the five Cs and presents distinct strengths and trade-offs for developing functionally effective, ecologically robust, and commercially translatable SynComs (Figure 1 and Table 1).

**FIGURE 1:**
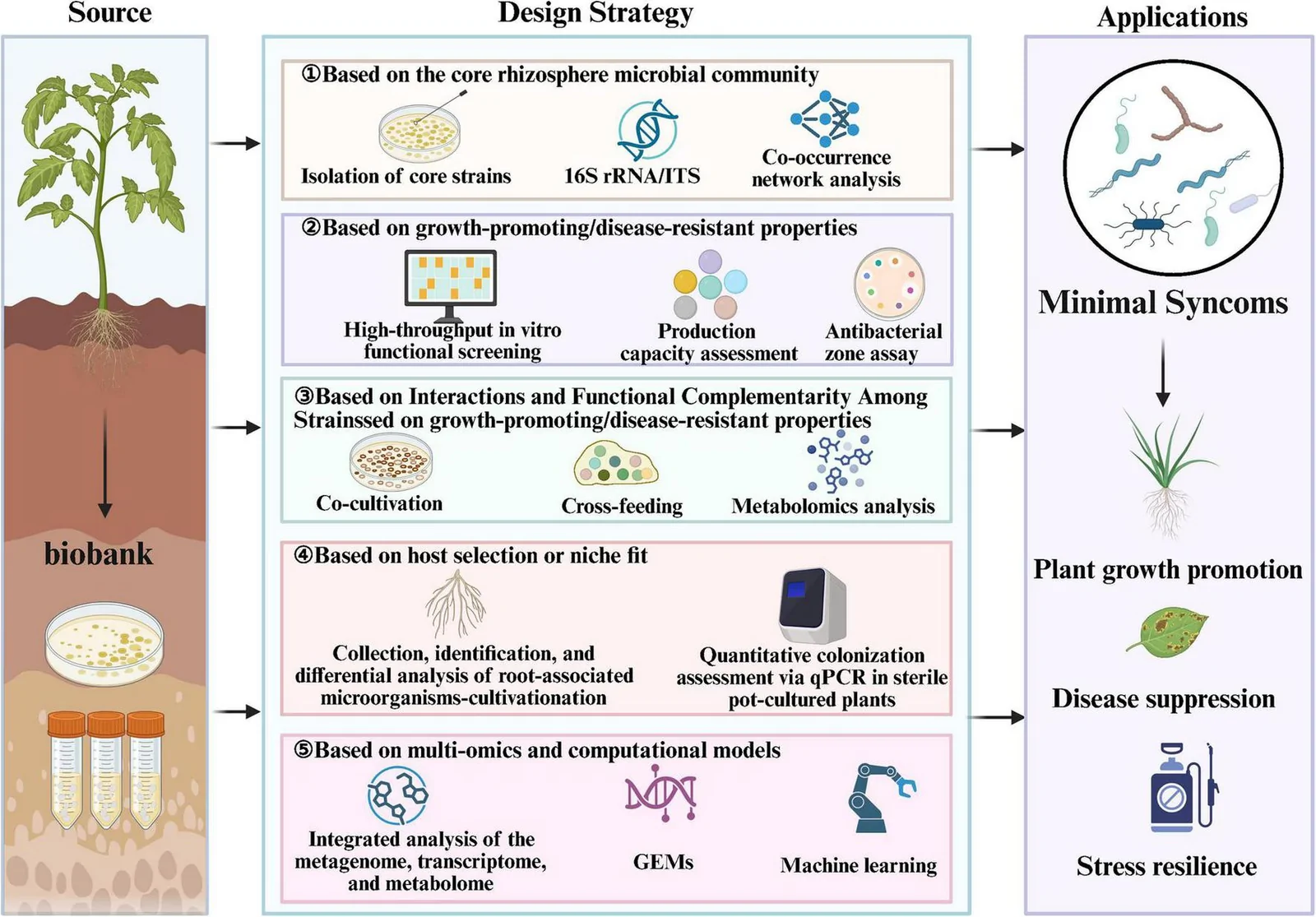
Strategies for constructing synthetic microbial communities and rational design workflows. SynComs can be constructed from plant-associated microbial resources through core microbiota-based selection, functional screening, strain-strain interaction analysis, host- or niche-adapted assembly, and multi-omics- or computational model-guided rational design. These approaches support the development of minimal and functionally stable SynComs for plant growth promotion, soil-borne disease suppression, and reduced dependence on chemical pesticides. Created in BioRender. Cai, W. (2026), https://BioRender.com/fmk28i2. BioRender Academic License, Agreement No. W7GZZXHSMB.

**TABLE 1:** Comparative assessment of major strategies for constructing plant-associated synthetic microbial communities.

Design strategy	Primary inputs	Immediate output	Main strengths	Main limitations	Principal five-C dimensions and evidence status
Core microbiome mining	Cross-sample 16S rRNA/ITS profiles; metagenomics; healthy-versus-diseased comparisons; co-occurrence analysis; culture collections	Candidate pool of persistent or health-associated taxa and isolates	High ecological relevance; may enrich for host-associated and persistent members; reduces the initial search space	Prevalence or abundance does not establish causal function; culture recovery can be biased; rare but important taxa may be missed	Primarily informs colonization and controllability. Supported by multiple plant proof-of-concept studies, but transferability remains system-dependent
Trait-based screening	Isolate libraries; plant-growth-promotion assays; antagonism assays; enzyme and metabolite measurements; stress-tolerance tests	Functionally characterized strains selected for defined target traits	Transparent selection criteria; experimentally accessible; scalable for initial screening; supports targeted functional design	*In vitro* traits may not be expressed *in planta* or in soil; ecological establishment and inter-strain interactions are often underrepresented	Primarily informs complementarity and controllability. Widely used *in vitro* and in pot studies, but field predictability remains limited
Interaction-informed assembly	Co-culture assays; pairwise interaction matrices; cross-feeding tests; metabolomics; biofilm assays; interaction networks	Combinations predicted or demonstrated to be compatible and functionally complementary	Directly addresses coexistence, emergent community function, metabolic exchange, and antagonism among members	Pairwise interactions may not predict higher-order dynamics; outcomes depend strongly on medium and environmental conditions	Primarily informs compatibility and complementarity. Mechanistic evidence is increasing, but higher-order and field validation remain limited
Host- and niche-specific filtering	Target-host genotype; root exudates; chemotaxis assays; attachment and biofilm tests; qPCR-based colonization assays; target soil conditions	A consortium tailored to a particular host genotype, tissue, soil, or environmental niche	Directly addresses ecological fit, establishment, persistence, and context-specific functional expression	High host and soil specificity may reduce transferability; screening is time- and resource-intensive	Primarily informs colonization and compatibility. Ecologically well supported, but conclusions may not generalize beyond the tested host-soil context
Multi-omics- and model-guided prioritization	Genomes; metagenomes; transcriptomes; metabolomes; phenotypes; environmental metadata; GEMs; stoichiometric models; machine learning	Ranked candidate combinations, predicted metabolic exchanges, and potentially minimal SynComs	Integrates multiple data types; reduces the combinatorial search space; supports mechanistic hypothesis generation and minimal-community design	Sensitive to annotation and modeling assumptions; incompletely represents spatial, regulatory, host-mediated, and resident-microbiome interactions; limited transferability across systems	Primarily informs complementarity and controllability, with potential commercial benefits through simplification. Plant-soil evidence remains largely proof-of-concept

The five-C dimensions indicate which criteria are principally informed by each strategy; they should not be interpreted as criteria that are automatically satisfied. Colonization, compatibility, functional performance, and commercial feasibility require independent experimental validation.

### 2.1 Core microbiome mining for candidate discovery

The rhizosphere core microbiota refers to microbial taxa that are consistently present at high abundance in the rhizosphere of specific crops and are closely associated with host health; these taxa exhibit host specificity, environmental stability, and functional conservation, making them a natural and high-quality resource pool for SynCom construction (Stopnisek and Shade, 2021). Constructing SynComs based on the rhizosphere core microbiota follows a nature-inspired design principle, which can substantially enhance the colonization efficiency and environmental adaptability of exogenous communities in the rhizosphere while reducing the risk of competitive exclusion by native soil microorganisms (Zhou et al., 2024).

The core workflow of this strategy proceeds as follows: first, the structure of the rhizosphere microbial community of healthy crops is characterized via 16S rRNA/ITS high-throughput sequencing, metagenomics, and co-occurrence network analysis to identify core microbial taxa; core strains are then isolated using culture-dependent techniques, and their key functions-such as growth promotion and disease suppression-are confirmed through genomic analysis combined with *in vitro* functional validation; finally, based on the species composition and interaction patterns of the core microbiota, key strains are selected to construct a simplified SynCom that retains core functions while reducing community complexity (Cao et al., 2024; Chukwuneme and Babalola, 2025; Fouad et al., 2025). For example, 16 core bacterial strains were screened from the rhizosphere of grafted watermelon to construct a SynCom, demonstrating that the SynCom markedly enhanced the resistance of non-grafted watermelon to *F. oxysporum*, with stronger colonization ability in natural soil than that of individual strains (Qiao Y. et al., 2024). Despite its ecological relevance, core microbiota-based SynCom design does not necessarily guarantee functional efficacy, because abundant taxa are not always functionally indispensable. Core taxa should be integrated with genome-resolved functional annotation, culture-based validation, and colonization assays before being prioritized for SynCom assembly.

### 2.2 Trait-based screening for growth-promoting and disease-suppressive functions

Trait-based screening is one of the most widely used strategies for constructing plant-beneficial SynComs. This approach selects microbial isolates according to measurable phenotypes related to plant growth promotion, stress tolerance, or pathogen suppression, and then combines functionally promising strains into defined communities (Zhou et al., 2026b). It offers strong targeting capacity, clearly defined functionality, and high design flexibility, allowing functional strains to be screened from diverse habitats including soil and plant endophytic environments, and is particularly suitable for the directed construction of highly efficient growth-promoting or disease-suppressive SynComs.

Strain screening is centered on two core dimensions: growth-promoting function and disease-suppressive function. Growth-promoting screening primarily targets strains capable of nitrogen fixation, phosphate/potassium solubilization, indole-3-acetic acid (IAA) secretion, and siderophore production, whereas disease-suppressive screening focuses on strains exhibiting strong antagonistic activity against soil-borne pathogens such as *F. oxysporum* and *R. solanacearum*, with screening criteria including the diameter of inhibition zones, cell-wall-degrading enzyme activity, and the capacity to secrete antimicrobial compounds (Timofeeva et al., 2023; Dai et al., 2025). For example, a research team at China Agricultural University screened three antagonistic strains–*Bacillus*, *Pseudomonas*, and *Talaromyces*–through *in vitro* antagonism assays, and the resulting SynCom exhibited significantly greater inhibitory effects against *R. solanacearum* than any single-strain alone (Yu et al., 2026).

### 2.3 Interaction-informed assembly and functional complementarity

Interaction-informed assembly is central to rational SynCom design because the performance of a microbial community is determined not only by the traits of individual members but also by their interactions. These interactions may include metabolic cross-feeding, resource partitioning, signaling-mediated coordination, biofilm cooperation, or cooperative pathogen inhibition. Conversely, strong antagonism, overlapping resource requirements, or incompatible growth dynamics may destabilize the community and reduce its functional output (Sun et al., 2023). The goal of interaction-informed design is to identify combinations of strains that are not only individually beneficial but also ecologically compatible and functionally complementary when assembled as a community.

Analysis of strain-strain interactions is a prerequisite for this strategy and can be achieved through cross-feeding assays, co-culture experiments, and metabolomic analysis to dissect the metabolic interaction networks among strains-for instance, metabolites secreted by one strain may serve as nutrient sources for another, or multiple strains may cooperatively secrete antimicrobial compounds or degrade pathogen cell walls. Co-culture experiments are also essential for excluding strains with strong antagonistic relationships, preventing intra-community competition that could compromise overall functionality.

Beyond strain identity and functional complementarity, SynCom performance may also depend on inoculum dose and the relative abundance of individual members. For example, *Trichoderma* strains capable of degrading pathogen cell walls can be combined with IAA-producing *Pseudomonas* strains to construct a SynCom with both growth-promoting and disease-suppressive functions (Zhou et al., 2026a). In addition, functionally redundant strains can be incorporated to enhance community structural stability (Ge, 2026): when the activity of one strain declines due to environmental fluctuations, redundant strains can compensate for the lost function, helping maintain community performance. Greater community richness does not necessarily lead to improved function, because additional strains may introduce resource competition or destabilizing interactions. Community size, inoculation dose, and strain ratios should be optimized experimentally rather than assuming that equal proportions or greater diversity are universally beneficial.

### 2.4 Host-and niche-specific filtering for ecological fit

Host genotype and rhizosphere-specific ecological niches are key constraints determining the colonization capacity and functional expression of exogenous microbial communities (Negre Rodríguez et al., 2025). Different crop cultivars exhibit substantial divergence in root exudate composition and root surface physicochemical properties, leading to marked differences in microbial recruitment and colonization preference. By directionally screening strains adapted to specific host characteristics for SynCom construction, this strategy enables precise matching with the rhizosphere ecological environment, substantially alleviates competitive exclusion between exogenous communities and native soil microorganisms, and addresses the root cause of inconsistent field performance of microbial inoculants. Centered on host–microbiome interactions, this strategy integrates multiple complementary screening systems, including comparative screening across crop cultivars, root-exudate-induced enrichment, chemotaxis-based strain screening, and quantitative evaluation of rhizosphere colonization ability, to achieve a high degree of compatibility between the SynCom and the rhizosphere niche (Pacheco-Moreno et al., 2024).

Core microbiome mining and host- or niche-adapted design are related but analytically distinct. The former identifies candidate taxa on the basis of persistence, prevalence, or association with host health, whereas the latter evaluates whether candidate isolates can establish and express their functions in a specified host genotype, tissue, soil, or environmental context. In practice, the two approaches can be applied sequentially: core microbiome analysis narrows the candidate pool, after which host- and niche-specific assays provide ecological filtering and validation. This strategy first compares rhizosphere microbial community composition across different crop cultivars to identify beneficial microbial taxa specifically enriched in resistant or high-yielding genotypes; root exudates from the target crop are then collected as chemotactic substrates, and chemotaxis-based screening is performed using chemotaxis plates or microfluidic devices to preferentially retain strains exhibiting strong chemotactic responses toward root-derived signaling molecules; multiple rounds of rhizosphere colonization assessment are subsequently conducted via quantitative PCR in axenic pot experiments and observation of root-surface biofilm formation to eliminate poorly colonizing strains; finally, strains exhibiting superior chemotactic ability, root-surface attachment capacity, and growth-promoting/disease-suppressive function are combined to form a dedicated SynCom tailored to the rhizosphere microenvironment of the target crop (Kwak et al., 2018). Previous research has shown that fusaric acid (FA) secreted by *Fusarium* can alter tomato root exudates, leading to significantly increased secretion of α-tomatine, tryptophan, rutin, 2-hydroxyglutaric acid, tyramine, and leucine in disease-resistant cultivars (López-Díaz et al., 2018; Nakayasu et al., 2021; Mattei et al., 2026). These compounds promoted the growth and biofilm formation of the disease-suppressive strain *Sphingomonas* sp. Sm12. This study revealed substantial differences in the capacity of resistant versus susceptible cultivars to recruit beneficial rhizosphere microorganisms following pathogen infection, providing a theoretical basis for designing more effective SynComs through host-genotype-based screening (Jin et al., 2024).

### 2.5 Multi-omics- and model-guided rational design

The integration of multi-omics data and computational modeling is accelerating the transition of SynCom construction from empirical strain combination toward more rational and predictive community design (Mariam et al., 2025). This approach relies on metagenomics, transcriptomics, and metabolomics to characterize the genetic potential, expression profiles, and metabolic cross-feeding relationships among strains, combined with genome-scale metabolic models (GEMs) and machine learning algorithms to quantitatively predict community compatibility, functional synergy, and environmental stability. This integrated strategy lowers the cost associated with *in vitro* trial-and-error approaches and enables the directed selection of optimal community combinations possessing both growth-promoting and disease-suppressive potential, overcoming the inherent limitation of single screening approaches in predicting strain antagonism and metabolic redundancy (Kimotho and Maina, 2024; Wu et al., 2024).

A typical multi-omics-guided workflow begins with the identification of candidate taxa or isolates from plant-associated microbiomes. Metagenomics is used to characterize the overall functional gene repertoire of the rhizosphere microbiome, identifying strains associated with nutrient mobilization, antimicrobial compound biosynthesis, and host immune induction; transcriptomic and metabolomic analyses are then employed to clarify gene expression and metabolite secretion patterns of strains under root-exudate induction, elucidating metabolic complementarity and cross-feeding relationships among strains. Genome-scale metabolic models for individual strains are subsequently constructed to simulate overall community metabolic flux under varying carbon and nitrogen conditions, allowing prediction of potential resource competition, mutualism, or mutual inhibition within the community. However, genome-scale metabolic models depend on genome-annotation quality, modeling assumptions, and the simulated nutrient environment. Accordingly, they generally predict feasible metabolic exchanges rather than realized fluxes, stable coexistence, or agronomic performance.

Finally, machine learning algorithms are introduced to integrate multi-omics high-throughput data, *in vitro* functional phenotypes, and pot-based disease-suppression and growth-promotion outcomes, enabling the selection of a minimal SynCom that retains structural stability and functional synergy while reducing the number of community members and lowering the burden of field colonization (De la Vega-Camarillo et al., 2026; Yadav et al., 2026). Machine-learning predictions are also constrained by limited and heterogeneous training datasets and may not transfer reliably across plant genotypes, soil types, or experimental systems. In this context, computational tools do not replace experimental validation; rather, they narrow the search space and generate testable hypotheses for SynCom assembly (Adhikary et al., 2026). Researchers used genome-scale metabolic models (GEMs) of 193 *Arabidopsis* rhizosphere bacterial strains, simulating metabolic flux under different carbon and nitrogen conditions via the SMETANA algorithm to predict metabolic complementarity and cross-feeding relationships within the community; using the Miscoto tool to screen for minimal combinations of GEMs, they found that as few as 2–3 strains were sufficient to complete specific metabolic pathways through metabolic exchange, revealing the relationship between functional redundancy and metabolic synergy (Mataigne et al., 2022).

These five strategies are complementary rather than mutually exclusive. Core microbiome mining and trait-based screening support candidate discovery, interaction-informed assembly evaluates compatibility and emergent properties, host- and niche-specific filtering improves ecological fit, and multi-omics and computational modeling prioritize combinations for experimental testing. No single strategy can independently satisfy all five Cs; robust SynCom design requires an iterative framework integrating candidate discovery, interaction testing, ecological validation, and deployment-oriented assessment (Table 1).

## 3 Mechanisms of SynCom-mediated plant growth promotion

### 3.1 Nutrient mobilization through division of labor

In SynComs, nutrient mobilization is not merely the sum of individual strain traits. Functional partitioning among nitrogen-fixing bacteria, phosphate-solubilizing bacteria, potassium-solubilizing bacteria, and helper strains may improve nutrient availability through cross-feeding, pH modulation, and functional redundancy. However, whether these interactions persist under field nutrient heterogeneity remains insufficiently resolved (Liang et al., 2022). A key factor limiting crop yield in agricultural soils is that the majority of nitrogen, phosphorus, and potassium exists in mineral- or organic-bound forms that are not directly accessible to plant roots. Through coordinated functional division of labor among community members, SynComs can perform biological nitrogen fixation and mineralize sparingly soluble phosphorus and potassium compounds, thereby enhancing the availability of readily absorbable nutrients in the rhizosphere and fundamentally improving the nutritional status of host plants (Hao et al., 2024).

To facilitate nitrogen fixation, SynComs typically combine free-living and associative nitrogen-fixing bacteria. Nitrogen-fixing strains convert atmospheric, chemically inert nitrogen gas into ammonium via nitrogenase activity, while non-fixing companion strains supply polysaccharides and organic acids as carbon sources and help alleviate oxygen-mediated inhibition of nitrogenase, resulting in overall nitrogen-fixing efficiency that substantially exceeds that of single-strain (Fouad et al., 2026). As an example of a defined co-inoculation rather than a rationally designed SynCom, Jabborova et al. (2021) reported that co-inoculation of *Bradyrhizobium japonicum* USDA110 with *Pseudomonas putida* NUU8 enhanced root and shoot growth, nodulation, and nitrogen-fixing capacity in soybean.

Multiple strains drive phosphate solubilization synergistically through the coordinated secretion of phosphatases and organic acids. A SynCom made up of plant growth-promoting bacteria isolated from Jala corn significantly boosted corn growth and yield. The SynCom increased field yields from 11 tons per hectare to 16 tons and reduced rust incidence from 12.5% to 8%, showing its biotech potential in sustainable farming (De la Vega-Camarillo et al., 2023).

With respect to potassium solubilization, certain strains secrete metabolites that serve as carbon sources for companion strains, which in turn secrete complementary metabolites such as citric acid in substantial quantities; this metabolic division of labor between paired strains constitutes the core mechanism underlying efficient potassium solubilization. It has been reported that individual application of *Pseudomonas nitroreducens* or *Pseudomonas monteilii* showed limited potassium-solubilizing efficiency due to soil pH constraints and competition from native microorganisms, leading to poor field stability and weak environmental tolerance. By contrast, a binary SynCom composed of these two strains enhanced available potassium release through synergistic potassium solubilization driven by metabolic complementarity (Gu et al., 2025).

### 3.2 Growth-regulating metabolites and stress hormone modulation

In addition to nutrient mobilization, SynComs can influence plant growth by producing growth-regulating metabolites or modulating stress-related hormonal pathways. Community members may produce phytohormones such as indole-3-acetic acid (IAA) and gibberellins, express enzymes such as 1-aminocyclopropane-1-carboxylate (ACC) deaminase, or release siderophores and other metabolites that indirectly affect plant development and stress adaptation. These functions can influence root system architecture, ethylene homeostasis, micronutrient acquisition, and plant tolerance to abiotic stress. However, metabolite-mediated effects are highly dose- and context-dependent; therefore, higher metabolite production does not necessarily translate into stronger plant growth promotion under all conditions (Yadav et al., 2025).

As a key hormone regulating primary root elongation and lateral root development, IAA biosynthesis within SynComs relies on functional division of labor: certain strains convert tryptophan into IPyA, while other strains perform the subsequent dehydrogenation step, and this synergistic mechanism enables IAA yields that far exceed those of individual strains. In one maize study, a compatible bacterial consortium showed higher IAA production than the individual strains and was associated with increased lateral root formation (Molina-Romero et al., 2017). However, such metabolite production and plant responses are strongly dependent on culture conditions, host genotype, and microbial composition.

1-Aminocyclopropane-1-carboxylate deaminase cleaves the ethylene precursor ACC, preventing excessive ethylene accumulation that would otherwise cause premature leaf abscission or root senescence under stress conditions, and thus represents a key mechanism underlying stress-resistant growth promotion. Given the substantial variability in enzymatic activity among wild-type single-strain, SynComs achieve functional complementarity by incorporating strains with high ACC deaminase activity alongside acid-producing strains that improve rhizosphere conditions to stabilize enzymatic performance. A previous study reported that a two-strain consortium reduced ethylene emission and improved growth in salt-stressed French bean. However, these effects were observed under controlled pot conditions with sterilized substrate and should therefore be interpreted as study-specific rather than representative of field performance. Further validation across soils, genotypes, and field environments is needed (Gupta and Pandey, 2019). However, these metabolite-mediated effects are strongly context-dependent, and their robustness across soil types, host genotypes, and field environments remains insufficiently established.

### 3.3 Rhizosphere habitat modification and abiotic stress resilience

After successful colonization of the root surface and surrounding rhizosphere soil, SynComs can enhance plant performance by modifying the rhizosphere habitat and improving host tolerance to environmental stress (Pradhan et al., 2022). These effects are mediated by changes in soil physicochemical properties, reorganization of resident microbial communities, and modulation of plant stress responses (Li et al., 2026). For example, extracellular polysaccharides produced by SynCom members may promote soil aggregation and improve water- and nutrient-holding capacity, whereas organic acids can regulate rhizosphere pH and alter mineral availability or toxicity. In a field study, an aluminum-tolerant root-associated community reduced exchangeable soil Al and improved rice yield under acidic-soil conditions (Liu et al., 2023). These responses nevertheless require validation across additional soil types, cultivars, and environments.

With respect to continuous-cropping obstacles, SynComs leverage interspecific interactions and chemotactic effects to selectively recruit beneficial native microorganisms while suppressing soil-borne pathogens, thereby restoring microecological balance disrupted by continuous monoculture (Zhang et al., 2024). Prior studies have shown that continuous cropping leads to the accumulation of *F. oxysporum*, accompanied by the enrichment of beneficial microbial communities. The research team innovatively developed a “directional antagonistic SynCom” Experimental results showed that the SynCom not only effectively suppressed the activity of *F. oxysporum* but also promoted root growth in lilies (Qin et al., 2026). Under drought stress, inoculation with a SynCom reduced yield losses in hybrid maize and promoted the recruitment of beneficial rhizosphere microorganisms (Armanhi et al., 2021). Similarly, a desert-rhizosphere-derived SynCom improved tomato growth and physiological stress responses under salinity in the presence of a resident soil microbiome (Schmitz et al., 2022).

Together, these studies indicate that SynComs can function as ecological modifiers of the rhizosphere rather than merely as providers of individual growth-promoting traits. By linking soil chemical regulation, microbial community remodeling, and host stress physiology, SynComs may enhance plant resilience to adverse edaphic and climatic conditions. Nevertheless, these effects are strongly context-dependent, and their persistence across soil types, host genotypes, and field environments requires further validation (Figure 2).

**FIGURE 2:**
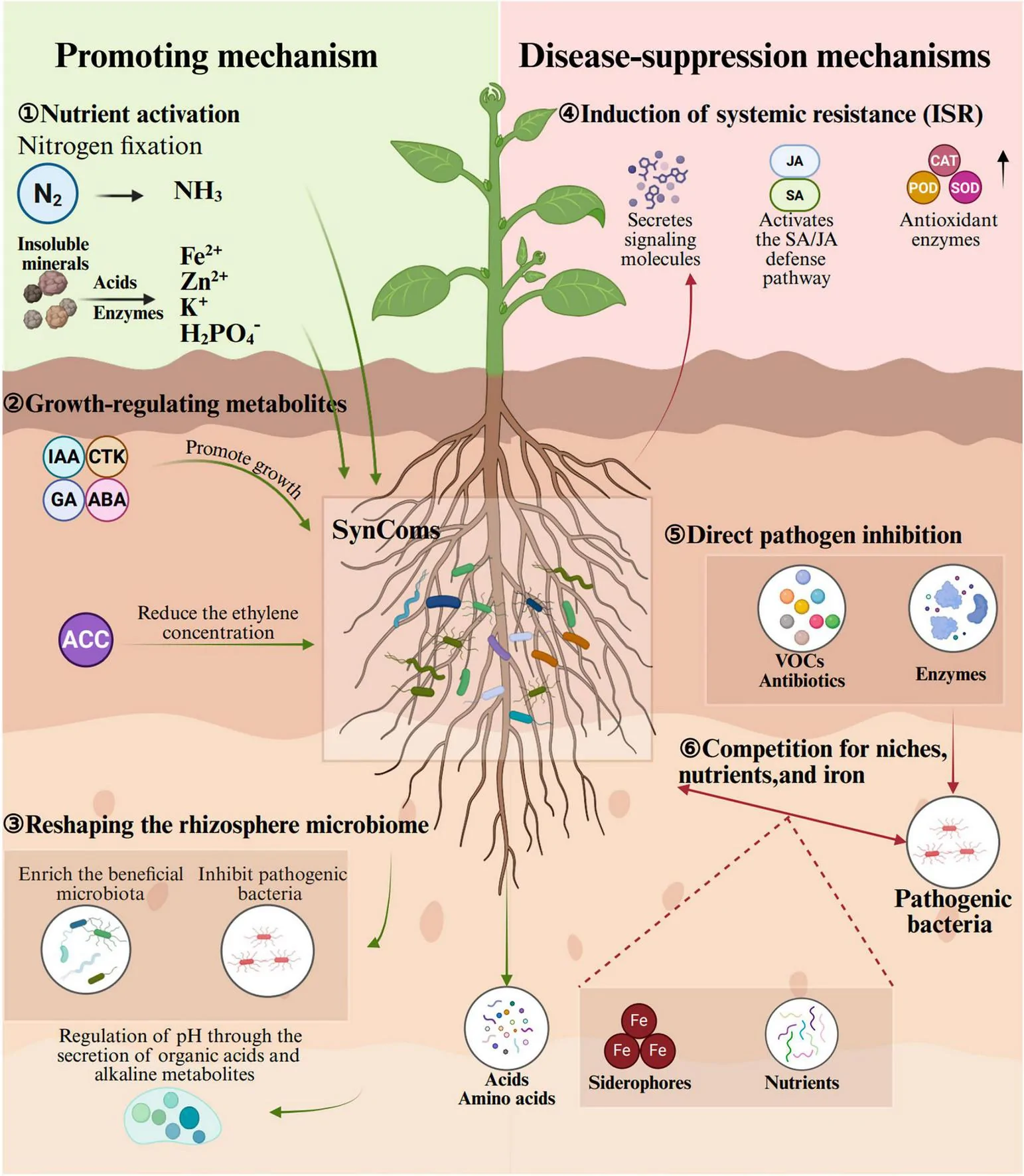
Integrated mechanisms of synthetic microbial communities in promoting plant growth and controlling soil-borne diseases. SynComs promote plant growth by mobilizing nutrients, producing growth-regulating metabolites, modulating ethylene levels, improving rhizosphere pH, and reshaping beneficial microbial communities. They suppress soil-borne diseases through nutrient and niche competition, siderophore-mediated iron sequestration, production of antibiotics, volatile organic compounds and hydrolytic enzymes, and activation of plant immune responses such as ISR through SA/JA signaling and antioxidant enzyme pathways. Created in BioRender. Cai, W. (2026), https://BioRender.com/4a1fb3k. BioRender Student Plan – Academic license, Agreement No.24SFYM3H6R.

Overall, quantitative effects reported across these studies should be interpreted cautiously because they were obtained under different host genotypes, soil conditions, inoculum regimes, stress treatments, and experimental systems. Controlled pot and greenhouse studies provide important mechanistic evidence, but their effect sizes should not be assumed to represent field performance. Independent validation across soils, genotypes, and field environments is therefore required to establish the robustness of SynCom-mediated growth promotion.

## 4 Mechanisms underlying soil-borne and soil-associated plant diseases suppression

### 4.1 Niche pre-emption and resource competition

The successful germination, colonization, and infection of plant roots by soil-borne pathogens depend critically on small-molecule carbon sources, iron, and root-surface attachment sites supplied by root exudates (Hao et al., 2012). Owing to their strong colonization capacity and diverse resource-utilization strategies, SynComs can establish a dual line of defense based on nutrient acquisition and niche occupation, which can limit pathogen germination through carbon and nitrogen depletion such as *Fusarium* spp., *R. solanacearum*, and *Rhizoctonia solani* (Table 2).

**TABLE 2:** Representative applications of SynComs and related defined microbial consortia in crop disease management.

Disease	Crop	SynCom components	Key mechanisms	Study-reported outcomes	References
Bacterial wilt	Tobacco	*Pseudomonas nicosulfuronedens*, *Pseudomonas citronellolis*, and *Bacillus altitudinis*	Chemotaxis; niche competition; antibiosis	Reduced the bacterial wilt disease index and increased tobacco yield	Liu Y. et al., 2025
Bacterial wilt	Tomato	*Alcaligenes faecalis*, *Stenotrophomonas maltophilia*, and *Leucobacter komagatae*	Antibiosis; induced systemic resistance	Suppressed *R. solanacearum* colonization; biocontrol efficacy was >45% higher than that of single-strain inoculation	Tanaka et al., 2025
*Fusarium* wilt	Banana	*Pseudomonas* sp. PS5, *Bacillus* sp. BN8.2, and *Trichoderma* sp. T2C1.4	Antibiosis; induced resistance; multi-target suppression	Reduced disease incidence and severity after inoculation	Prigigallo et al., 2022
*Fusarium* wilt	Cowpea	*Bacillus velezensis* HAB-2, *Pseudomonas hunanensis*, and *Enterobacter soli*	Defense enzyme activation; PR gene induction; synergistic antagonism	Higher resistance-related responses than the corresponding control	Wei et al., 2025
*Fusarium* wilt	Cucumber	*B. velezensis*, *Paenibacillus polymyxa*, and *B. subtilis*	Functional complementarity; cooperative metabolism; consortium stabilization	Enhanced antagonistic activity and improved overall biocontrol potential	Yang et al., 2025
*Fusarium* wilt	Tomato	*B. velezensis* and *Trichoderma guizhouense*	Bacteria-fungus compatibility; rhizosphere colonization; immune activation	Increased beneficial microbial colonization, activated immunity, and established a biological barrier	Li et al., 2025
Early blight	Tomato	*B. subtilis* J3 and *P. fluorescens* J8	Antibiosis; growth promotion; microbiome modulation	Reported pathogen inhibition of up to 94.91% under the tested conditions	Jia et al., 2023
Root rot	Wheat	*Pseudomonas* sp. B5, *Pseudomonas* sp. B11, *Pseudomonas* sp. B12, and *Pseudomonas* sp. P25	Nutrient competition; root-surface barrier; community-level suppression	Reduced root rot severity caused by *R. solani* AG8 and increased root weight	Yin C. et al., 2022
Root rot	Pepper	*Bacillus* sp. T3, *Flavobacterium anhuiense* T4, *Cytobacillus firmus* T8, *Streptomyces roseicoloratus* T14, and *Pseudomonas frederiksbergensis* A6	P solubilization; IAA/siderophore production; microbiome remodeling	Improved plant growth traits and activated nitrogen-cycling and pathogen-suppression pathways	Bashizi et al., 2025
*Fusarium* wilt and root rot	Apple	*Bacillus thuringiensis*, *B. velezensis*, *Bacillus siamensis*, *Bacillus toyonensis*, *B. cereus*, *Bacillus mycoides*, and *B. subtilis* J-28	Antibiosis; rhizosphere bacterial enrichment	Increased rhizosphere bacterial richness, enriched putatively beneficial genera, and suppressed pathogenic fungi	Qiao R. et al., 2024
*Fusarium* wilt and root rot	Strawberry	*P. putida* J10, *Pseudomonas aeruginosa* J9, *Pseudomonas stutzeri* J29, *P. stutzeri* J32, and *P. stutzeri* J13	Beneficial microbiome enrichment; protist diversity; pathogen suppression	Suppressed pathogenic fungi over six cropping generations and produced positive legacy effects on biomass and disease resistance related metabolism	Lei et al., 2026

Outcomes are study-reported and may not be directly comparable across studies; the predominance of positive findings may partly reflect publication and selection bias.

With respect to nutrient competition, strains within a SynCom possess differentiated carbon-source metabolic profiles, enabling rapid degradation of sugars, amino acids, and organic acids released by plant roots (Fonseca-García et al., 2024). This accelerated depletion of readily available nutrients markedly reduces carbon and nitrogen availability in the rhizosphere, which can limit pathogen germination through carbon and nitrogen depletion (Gómez Expósito et al., 2017). A study using a highly diverse bacterial community assembled as a SynCom to suppress root rot demonstrated that high bacterial diversity inhibits pathogens through mechanisms potentially including nutrient competition and the formation of physical barriers on the root surface (Bai et al., 2026). In addition, research has shown that, under iron-deficient soil conditions, beneficial microorganisms can secrete specific siderophores to chelate environmental iron, thereby restricting pathogen growth; moreover, the greater the intensity of siderophore-mediated competitive interactions within the community, the stronger the resulting resistance of the SynCom to pathogen invasion (Shao et al., 2024).

Niche pre-emption by SynComs is achieved through preferential attachment to plant roots, where dense biofilms are formed to seal off pathogen entry routes and root wound sites, establishing a robust physical barrier. One study reported that SynComs constructed on the basis of core strains can significantly enhance the biofilm-forming capacity of the entire microbial community, which may contribute to improved plant health and stress resistance (Liu X. et al., 2025).

### 4.2 Antimicrobial metabolites, VOCs, and hydrolytic enzymes

Through functional division of labor among community members, SynComs synthesize three major classes of antimicrobial products-lipopeptide antibiotics, volatile organic compounds (VOCs), and cell-wall-degrading hydrolytic enzymes-contribute to broad-spectrum disease suppression through additive or synergistic metabolic effects (Carrión et al., 2019). In contrast to individual strains, which often produce only a limited range of antimicrobial compounds, coordinated community action enables the simultaneous release of multiple classes of active substances, thereby suppressing pathogen growth through multiple concurrent pathways, including disruption of pathogen cell membranes, inhibition of spore germination, and degradation of fungal cell walls (Minchev et al., 2021).

Representative antibiotic metabolites include *Bacillus*-derived lipopeptides, such as fengycin, iturin, and surfactin, as well as *Pseudomonas*-derived 2,4-diacetylphloroglucinol (DAPG) and phenazines. Different microbial taxa may perform distinct functional roles within SynComs. For example, *B. velezensis* primarily produces lipopeptides that disrupt fungal membrane permeability (An et al., 2024), whereas *P. fluorescens* specializes in the synthesis of phenolic antibiotics that inhibit the proliferation of bacterial pathogens (Biessy and Filion, 2018). VOCs diffuse through the soil gas phase, enabling non-contact, long-range antimicrobial activity; one study demonstrated that multiple *Streptomyces* strains within a SynCom cooperatively release metabolites (such as inducing the production of erythromycin E and other active compounds), thereby substantially enhancing biocontrol efficiency against pathogens (Sun et al., 2025). Hydrolytic enzymes, including chitinase, β-glucanase, and amylase, are cooperatively secreted by *Pseudomonas, Bacillus*, and *Streptomyces* strains, targeting and degrading the chitin and glucan backbone of fungal cell walls, ultimately causing lysis of pathogen hyphae (Liu et al., 2019). However, broad-spectrum antimicrobial metabolites and hydrolytic enzymes may also affect non-target beneficial microorganisms, highlighting the need to evaluate pathogen suppression together with potential impacts on resident microbiota.

### 4.3 Immune priming and systemic resistance

Microbial cell components, extracellular secretions, and quorum-sensing signaling molecules produced by SynComs act as immune elicitors, activating induced systemic resistance (ISR) and systemic acquired resistance (SAR) in host plants. These responses involve coordinated regulation of the salicylic acid (SA), jasmonic acid (JA), and ethylene (ET) signaling pathways, leading to the upregulation of pathogenesis-related (PR) genes and antioxidant enzyme-encoding genes, thereby conferring broad-spectrum disease resistance not only in roots but also in aerial tissues that have not been directly exposed to pathogens (Ebrahimi-Zarandi et al., 2022; Jung et al., 2024).

Induced systemic resistance represents a core intrinsic mechanism underlying enhanced plant tolerance to both abiotic and biotic stress. Secondary metabolites and structural components of the SynCom act as elicitors that activate jasmonic acid and salicylic acid signaling, upregulating disease-resistance genes and antioxidant enzyme expression to confer broad-spectrum resistance. Li et al. demonstrated that *Bacillus cereus* AR156 primes camalexin biosynthesis to induce resistance against multiple pathogens, activating the SA and JA signaling pathways and substantially increasing the transcript levels of PR resistance genes (PR1, PR5); following pathway activation, the expression of antioxidant enzyme genes such as SOD and POD remained persistently upregulated (Li Z. J. et al., 2024).

### 4.4 Resident microbiome remodeling and disease-suppressive soil functions

The rhizosphere microbial community constitutes the first biological line of defense against soil-borne pathogen invasion, and the stability of its structure and function directly influences plant health. Prolonged continuous cropping or inappropriate agricultural management practices commonly disrupt rhizosphere microecological balance, manifesting as a decline in beneficial microbial diversity and an increase in pathogen dominance, which in turn triggers outbreaks of soil-borne disease (Zhang et al., 2025a,b,c). By introducing beneficial microorganisms that have undergone functional screening and combinatorial optimization into the rhizosphere, SynComs can actively reshape rhizosphere community structure and restore or enhance its disease-suppressive function. SynCom-based microbiome engineering represents a promising strategy for the management of soil-borne diseases (Wen et al., 2023).

The diversity of the rhizosphere microbial community is a key determinant of the stability of its ecological function. Studies have found that introducing a SynCom composed of multiple *Pseudomonas* strains into the tomato rhizosphere resulted in greater community diversity being associated with higher rhizosphere colonization survival and significantly stronger suppression of *R. solanacearum*, high-diversity communities reduced pathogen density and disease incidence by intensifying nutrient competition and niche occupation. An ecological mechanism whereby “probiotic diversity enhances rhizosphere microbiome function,” provides a theoretical framework for SynCom design (Hu et al., 2016).

Beyond enhancing diversity, SynComs can also selectively enrich beneficial taxa while suppressing pathogenic taxa, thereby reshaping rhizosphere community structure (Figure 2). Previous research has found that continuous monoculture of peanut caused key disease-suppressive microbial taxa in the rhizosphere to be outcompeted and depleted due to limited access to root exudates, significantly reducing the soil’s capacity to suppress root rot; inoculation with a SynCom reconstructed from these depleted native strains restored rhizosphere resistance to pathogens. From the perspective of niche competition, the mechanism by which SynComs restore soil disease-suppressive function (Zhou et al., 2023).

Current evidence does not support a universal hierarchy of disease-suppression mechanisms across SynCom systems. Resource and niche competition and direct antagonism have been repeatedly implicated across multiple crop–pathogen systems, whereas specific mechanisms such as VOC-mediated inhibition or biofilm-dependent exclusion are supported by more context-specific evidence. Immune priming and resident microbiome remodeling are also frequently associated with disease suppression but often occur together with direct microbial antagonism and competition, making their independent contributions difficult to resolve. SynCom-mediated disease suppression is best viewed as a context-dependent, multi-mechanistic process rather than the result of a single dominant mechanism.

## 5 Applications in crop–pathogen systems and continuous-cropping obstacles

### 5.1 Applications in the management of bacterial wilt, Fusarium wilt, early blight, and related diseases

Soil-borne and soil-associated diseases represent major biotic constraints on agricultural production. Among these, bacterial wilt caused by *R. solanacearum*, Fusarium wilt caused by *F. oxysporum*, and early blight induced by *A. solani* pose major challenges to global agricultural sustainability, owing to their complex pathogenic mechanisms and the considerable difficulty associated with their control (Meshram and Adhikari, 2024). As an emerging biocontrol strategy, SynComs integrate functional complementarity and synergistic interactions among multiple beneficial microorganisms, thereby demonstrating substantial application potential in the control of soil-borne diseases. Across the 11 representative crop–disease applications examined in this review, substantial heterogeneity was evident in crop and pathogen identity, SynCom composition, experimental setting, comparator, and outcome definition. Consequently, the reported effect sizes are not directly comparable and were not pooled. Where percentage-based outcomes were available, SynComs achieved more than 45% greater biocontrol efficacy than single-strain inoculation and up to 94.91% pathogen inhibition. However, many studies reported disease incidence, disease severity, pathogen abundance, plant biomass, or yield using different metrics, highlighting the need for more standardized outcome reporting and multi-site field validation (Table 1).

In managing bacterial wilt, conventional single-strain biocontrol agents often fail to maintain their antimicrobial efficacy due to limited colonization capacity and poor environmental adaptability (Chandrasekaran et al., 2016). A recent study demonstrated that a chemotaxis-based SynCom, screened on the basis of the chemotaxis gene cheA, could effectively suppress pathogen invasion through directed regulation of rhizosphere community structure. A chemotaxis-guided three-member SynCom reduced bacterial wilt severity and improved tobacco performance under field conditions (Liu Y. et al., 2025). To address the common loss of efficacy of single biocontrol strains caused by antagonism from native soil microorganisms, a strain-compatible SynCom was assembled. Its members performed three complementary functions: directly producing antimicrobial compounds, regulating host rhizosphere microecology, and inducing systemic disease resistance in host plants. Under both axenic-substrate and field-soil conditions, the SynCom significantly suppressed colonization by *R. solanacearum*, achieving more than a 45% improvement in disease-control efficacy relative to single-strain treatments (Tanaka et al., 2025).

With respect to Fusarium wilt control, the application of cross-kingdom SynComs has shown promising disease-suppressive potential. Studies have shown that SynComs incorporating multiple distinct strain types can suppress plant pathogens through complementary mechanisms, integrating the strong antagonistic activity of *Trichoderma* fungi with the induced-systemic-resistance functions of *Pseudomonas* and *Bacillus* strains. In pot experiments using steam-sterilized soil, an optimized SynCom (SynCom 1.2), composed of *Pseudomonas* sp. PS5, *Bacillus* sp. BN8.2, and *Trichoderma* sp. T2C1.4, significantly reduced both the incidence and severity of banana Fusarium wilt following inoculation (Prigigallo et al., 2022). However, these results should not be interpreted as field validation, and their robustness under non-sterile field conditions remains to be established. In addition, a study on cowpea Fusarium wilt confirmed that a three-strain biocontrol consortium comprising *B. velezensis* HAB-2, *P. hunanensis* and *E. soli* improve plant resistance to the pathogen by activating plant defense enzyme systems and upregulating pathogenesis-related gene expression (Wei et al., 2025). To manage cucumber Fusarium wilt, a research team combined three mutually compatible biocontrol strains: *B. velezensis*, *P. polymyxa*, and *B. subtilis*. The authors noted that while single strains are highly susceptible to environmental fluctuations and limited by a single mode of action, combining multiple strains enhances antagonistic efficacy through functional complementarity, thereby improving overall community stability and biocontrol potential (Yang et al., 2025).

The development and application of SynComs have shown considerable potential for controlling early blight caused by *A. solani* (Wang Z. et al., 2023). Recent studies have highlighted that a shift in bacterial–fungal interaction patterns, from antagonism to coexistence, plays a critical role in improving plant disease suppression. By screening mutant strains, the team successfully constructed a coexisting combination of *B. velezensis* and *T. guizhouense*. Inoculation with this cross-kingdom combination not only significantly increased the colonization abundance of beneficial microorganisms in the rhizosphere but also altered rhizosphere microbial community function, enhancing tomato immune responses and establishing an effective biological barrier. This study provides important theoretical and molecular mechanistic support for the future rational construction of functionally complementary SynCom (Li et al., 2025). Other studies have confirmed that combining multiple strains, relative to single biocontrol strains, can achieve multi-pathway synergistic control through functional complementarity: a SynCom formed by co-culturing *B. subtilis* J3 with *P. fluorescens* J8 achieved a pathogen inhibition rate as high as 94.91%, with the underlying synergistic mechanism involving not only direct pathogen suppression but also reduced activity of defense enzymes such as peroxidase in tomato plants, enhanced plant growth, and substantial restructuring of rhizosphere and phyllosphere microbial community structure, collectively achieving multi-pathway synergistic disease control (Jia et al., 2023).

In the control of root rot and other complex soil-borne disease complexes, SynComs demonstrate distinctive ecological regulatory advantages through the construction of highly diverse bacterial communities. Recent studies have revealed that high-diversity bacterial SynComs can effectively suppress root rot incidence through nutrient competition and the formation of physical barriers on the root surface. Rhizosphere-derived SynComs have shown particularly strong performance in controlling wheat root rot caused by *R. solani* AG8, with a four-strain *Pseudomonas consortium* (SynCom C1) significantly reducing disease severity and increasing root biomass (Yin C. et al., 2022). Further research on pepper Phytophthora root rot (caused by *Phytophthora capsici*) demonstrated that a five-strain SynCom–comprising *Bacillus* sp. T3, *F. anhuiense* T4, *C. firmus* T8, *S. roseicoloratus* T14, and *P. frederiksbergensis* A6–not only improved plant growth indices through growth-promoting functions such as phosphate solubilization, IAA production, and siderophore production, but also reshaped rhizosphere community structure, enriched beneficial genera, and activated functional pathways related to nitrogen cycling and pathogen suppression (Bashizi et al., 2025). Overall, disease-targeted SynComs appear to rely on combinations of chemotaxis, rhizosphere colonization, siderophore-mediated competition, direct antagonism, and ISR activation. However, reported effects are difficult to compare because experimental conditions and validation levels differ among studies. Of the nine disease-control case studies reviewed, only two included field validation, and none involved multi-site field testing, limiting conclusions about the consistency of SynCom performance across environments.

### 5.2 SynComs as microbiome restoration tools for continuous-cropping obstacles

Continuous-cropping obstacles represent a critical bottleneck limiting the sustainable productivity of intensive agricultural systems. These obstacles arise from the combined effects of multiple factors, including rhizosphere microecological imbalance, pathogen enrichment, accumulation of allelopathic autotoxic compounds, and deterioration of soil physicochemical properties. Prolonged monoculture of a single crop drives a shift in rhizosphere microbial community structure from bacteria-dominated to fungi-dominated assemblages, accompanied by a sharp decline in beneficial microbial diversity and a marked increase in the abundance of pathogenic fungal taxa, ultimately triggering recurrent outbreaks of soil-borne disease together with simultaneous declines in crop yield and quality (Gao et al., 2024; Xu et al., 2025). As a core strategy in microbiome engineering, SynComs-by introducing functionally well-defined and interactively coordinated beneficial microbial combinations into degraded soils-offer an innovative pathway for reconstructing soil microbial networks and restoring soil health functions.

Disease-suppressive soils represent a unique soil phenotype in which disease incidence is markedly suppressed despite the presence of both the pathogen and a susceptible host. In nature, long-term monoculture of wheat or barley can spontaneously give rise to soils suppressive to take-all disease, a phenomenon known as take-all decline (TAD). The core mechanism underlying TAD involves the directional enrichment of 2,4-diacetylphloroglucinol-producing strains within rhizosphere *P. fluorescens* populations. The classic studies of Raaijmakers and Weller demonstrated that the density of DAPG-producing strains in TAD soils can reach over 10^5^ CFU per gram of root, far exceeding the disease-suppressive threshold, and that this suppressiveness can be transferred to susceptible soils through inoculation with as little as 1%∼10% disease-suppressive soil. SynCom technology is grounded in this natural ecological principle, accelerating the induction of disease-suppressive soils through the artificial construction of SynComs containing key functional strains such as DAPG-producing bacteria, thereby circumventing the prolonged timescales required for natural soil suppressiveness to develop (Kwak and Weller, 2013).

Secondary metabolites secreted by crop roots-including phenolic acids, terpenoids, and alkaloids-accumulate progressively in continuously cropped soils, giving rise to autotoxicity that directly inhibits seed germination, root elongation, and nutrient uptake, while also selectively enriching pathogenic microorganisms and further exacerbating rhizosphere microecological deterioration (Bao et al., 2022). In potato monoculture systems, rhizosphere autotoxic compounds drive directional succession of the microbial community, leading to functional decline of beneficial microbial groups (Xin et al., 2022). Researchers constructed defined bacterial and fungal communities capable of degrading potato rhizosphere autotoxins and incorporated them into sheep-manure organic fertilizer to produce a bio-organic fertilizer (BOF). Pot experiments showed that, although this SynCom-based BOF formulation had only a limited effect on soil microbial α-diversity, it substantially altered community β-diversity, activated rare taxa associated with stress tolerance, nitrogen fixation, and saprotrophic functions, and reshaped the composition of key species within the microbial co-occurrence network. Notably, fungal SynComs exhibited markedly higher colonization efficiency in the organic fertilizer and soil (81.0%∼85.2%) compared with bacterial SynComs (14.4%∼20.4%), whereas combined bacterial-fungal SynComs more effectively enhanced fungal colonization rates in rhizosphere soil (Zhang et al., 2024).

By introducing exogenous beneficial microorganisms that engage in competitive, mutualistic, or niche-complementary interactions with native microbial communities, SynComs can reconstruct the process of community assembly. In a study on apple continuous-cropping obstacles, a SynCom composed of eight antagonistic strains, including *Bacillus* and *Streptomyces* species, not only significantly suppressed four pathogens–including *F. oxysporum* and *R. solani*–but also, after 84 days of colonization, significantly increased rhizosphere bacterial richness, elevated the relative abundance of potentially beneficial genera, and suppressed the abundance of pathogenic fungi. This study revealed that SynCom application can drive a shift in rhizosphere microbial community structure from a “fungi-dominated” to a “bacteria-enriched” state, closely paralleling the microbial signatures characteristic of disease-suppressive soils (Qiao R. et al., 2024).

Combining SynComs with soil amendments such as biochar and organic fertilizers can establish a tripartite “microbe-carrier-substrate” synergistic system that substantially enhances microbial survival and functional expression in continuously cropped soils. The porous structure of biochar provides a protective microhabitat for microorganisms, while its surface functional groups adsorb and immobilize autotoxic compounds, and its slow-release nutrient properties support sustained microbial metabolic activity. Studies have shown that biochar-based organic fertilizer significantly increased rhizosphere bacterial α-diversity in continuously cropped tobacco soil, enriching beneficial genera such as *Arthrobacter* and *Pseudomonas* while reducing the abundance of potentially pathogenic genera such as *Mycobacterium*; within the fungal community, the abundance of antagonistic fungal genera such as *Trichoderma*, *Mortierella*, and *Penicillium* increased, while pathogenic fungi were suppressed (Chen et al., 2025).

The beneficial effects of SynComs on soil health are not limited to a single growing season; colonizing communities can establish an ecological memory that continues to exert beneficial effects across subsequent cropping cycles. In a strawberry multi-generation continuous-cropping system, a SynCom composed of *Bacillus* and *Pseudomonas* strains significantly increased the Shannon diversity index of beneficial rhizosphere bacteria and consumer protist communities across six consecutive cropping generations, while continuously suppressing the diversity and relative abundance of the pathogenic fungus *F. oxysporum*. Remarkably, this SynCom continued to exert positive legacy effects on beneficial rhizosphere bacterial abundance, plant biomass, and disease-resistance-related metabolic pathways (such as phenylpropanoid biosynthesis and α-linolenic acid metabolism) in the seventh cropping generation, while maintaining a negative effect on pathogen abundance (Lei et al., 2026). This finding confirms that rationally designed SynComs can establish self-sustaining microbial regulatory networks in continuously cropped soils, overcome the technical bottleneck whereby conventional microbial inoculants lose efficacy in subsequent growing seasons, and provide a sustainable microbiome-based solution for the long-term management of continuous-cropping obstacles. Studies of continuous-cropping systems suggest that SynComs can reshape resident microbiomes and enhance disease-suppressive functions. However, their magnitude, persistence, and specific contribution remain difficult to compare across studies, particularly when combined with organic amendments.

## 6 Translational challenges and deployment strategies

The limitations highlighted throughout the preceding sections become particularly important when SynComs are translated to field application. Although SynComs have demonstrated broad potential for plant growth promotion and the management of soil-borne diseases, their translation from laboratory research to large-scale field application remains constrained by numerous bottlenecks. Current research has predominantly focused on functional validation under controlled conditions, while critical scientific questions concerning the stability, interaction mechanisms, and industrialization pathways of SynComs in complex agroecosystems remain insufficiently understood. A systematic examination of these constraints, together with a clear articulation of future directions, is therefore essential for advancing the maturation and practical deployment of SynCom technology.

### 6.1 Field stability and ecological establishment

Heterogeneity in field environments-including fluctuations in temperature, soil pH, nutrient availability, and water stress-can substantially affect the survival and functional expression of SynCom members. Studies have shown that some strains exhibiting outstanding performance during *in vitro* screening lose activity when they fail to adapt to the complex physicochemical conditions of field soils. This challenge is further intensified by biotic pressures such as competitive exclusion by native microorganisms, host immune responses, and phage predation, all of which substantially reduce the colonization success of SynComs (Zhang et al., 2026).

The instability of conventional microbial inoculants under field conditions has long been recognized, and its root cause lies in the difficulty inoculated strains face in successfully colonizing the rhizosphere amid competition from native microorganisms; the interaction of multiple factors-including soil type, climatic conditions, seasonal variation, and crop genotype-often results in highly variable field trial outcomes (Kleinbölting et al., 2026). Future research needs to move beyond the mere optimization of laboratory culture conditions and instead establish host–SynCom simulation systems that integrate *in situ* response monitoring with artificial intelligence-based modeling, enabling the prediction and optimization of SynCom adaptability within specific ecosystems. In parallel, innovative deployment strategies-such as carbon-based material encapsulation techniques for strain protection-should be explored to enhance the physicochemical tolerance and biological competitiveness of SynComs under field conditions.

### 6.2 Interaction mechanisms and higher-order community dynamics

The functional output of a SynCom is not simply the additive sum of the independent effects of its individual members, but instead emerges from a complex network of interspecific interactions, including metabolic complementarity, resource competition, and antagonism operating at multiple hierarchical levels. However, current understanding of these interaction mechanisms remains largely descriptive, lacking systematic dissection of higher-order interactions (Wang D. et al., 2023; Ishizawa et al., 2025).

At the level of metabolic interaction, cross-feeding represents a mechanism sustaining the coexistence of SynCom members. Studies employing co-evolution experiments with amino acid auxotrophic strains have demonstrated the emergence of mutualistic cycles: when one strain produces an excess of a metabolite required by its partner, the partner gains a competitive advantage and may subsequently release greater amounts of a metabolite required by the first strain. This reciprocal exchange can promote the growth of both partners and stabilize their coexistence. This cooperative relationship is highly context-dependent–under nutrient-rich conditions, cross-feeding can maximize the yield of a target metabolite, whereas the introduction of additional strains may give rise to emergent competitive interactions that instead reduce overall community efficiency (Jia et al., 2016).

At the level of antagonistic interaction, interspecific competition encompasses not only the contest for nutritional and spatial resources but also active suppression mediated by chemical weapons such as antibiotics, organic acids, bacteriocins, and biosurfactants. Recent studies have revealed that competitive outcomes are strongly influenced by the phylogenetic relatedness of strains and the degree of overlap among their biosynthetic gene clusters, with predictive power exceeding that of conventional plate-based inhibition assays. Under resource-rich conditions, antibiotics selectively target competitors that occupy similar ecological niches but possess divergent genetic backgrounds, whereas under nutrient-limited conditions, microorganisms tend to adopt alternative survival strategies rather than relying on antibiotic-mediated suppression (Wang et al., 2025).

### 6.3 Scaling-up Fermentation, product development, and quality control

The translation of SynComs from laboratory research to industrial-scale production faces multiple technical and economic bottlenecks.

Scaling up fermentation presents its own challenges. Outstanding performance observed in laboratory shake-flask cultures is often difficult to replicate directly in industrial-scale bioreactors. During large-scale fermentation, reduced gas-liquid mass transfer efficiency, increased shear stress, and uneven substrate distribution can lead to deterioration of strain metabolic performance or even cellular damage. Studies have indicated that the capital investment required for scaling up industrial fermentation facilities typically exceeds that required for strain and process development, and that the timeline from laboratory research to commercial-scale production is often considerable (Crater and Lievense, 2018).

Formulation stability remains a major hurdle, as the inherent biological constraints of living microorganisms often lead to short shelf lives and strict storage needs. Although recently developed spray-drying and cross-linked alginate microencapsulation techniques enable industrial-scale production, strain viability loss during spray drying can still reach 2.5∼3.0 log units. Extending room-temperature stability therefore requires the addition of protective agents such as trehalose and maltodextrin, together with controlled storage conditions (low oxygen and low humidity) (Arbaugh et al., 2022).

Unlike single-strain inoculants, SynCom scale-up must preserve both strain viability and community composition. Differential growth and stress tolerance among members can cause compositional drift during co-fermentation, making separate cultivation followed by controlled mixing a practical alternative. For example, a seven-strain maize SynCom was produced by culturing each strain independently, standardizing cell density, and mixing equal volumes before field application, thereby maintaining a defined starting composition (Hernández-García et al., 2025). However, this approach increases production complexity and does not by itself demonstrate industrial-scale batch consistency or shelf stability. Commercial-scale testing also illustrates this translational gap. In a 6-month hydroponic tomato trial, two three-member SynComs retained no adverse effects on final yield or fruit quality, but both transiently reduced early plant growth and only one introduced strain consistently persisted, despite stronger benefits under laboratory conditions (Wilkinson et al., 2026). This highlights that successful laboratory performance does not necessarily translate into stable community persistence or improved agronomic performance at larger scales.

Economically, the absence of unified quality certification systems and regulatory standards, together with limited farmer confidence in microbial products, continues to constrain market adoption. Accordingly, the development of low-cost, high-throughput fermentation platforms, the optimization of strain encapsulation and protection technologies, and the establishment of standardized scale-up pipelines from laboratory to field represent urgent priorities for advancing the industrialization of SynComs.

### 6.4 Biosafety, regulation, and environmental monitoring

Particular caution is required when SynComs contain taxa related to opportunistic pathogens, such as *P. aeruginosa*, *S. maltophilia*, or *B. cereus* groups. Strain-level genomic analysis, virulence-factor screening, antibiotic-resistance profiling, and non-target toxicity assessment should be conducted before field deployment. The ecological safety evaluation framework for field-deployed SynComs remains underdeveloped, and systematic quantification of long-term environmental risk is lacking (Gullì et al., 2025).

Following colonization, exogenous SynComs engage in continuous resource competition and metabolic interaction with native microorganisms; however, existing studies have predominantly focused on short-term community structural changes, whereas the persistence and ecological consequences of introduced strains across multiple growing seasons remain poorly resolved. Consequently, it remains unclear whether SynCom application can cause persistent depletion of beneficial native taxa, disruption of soil nutrient-cycling functions, or loss of disease-suppressive capacity. The non-target effects associated with broad-spectrum antimicrobial activity may also extend beyond immediate pathogen suppression and alter native microbial interaction networks, potentially affecting beneficial rhizosphere taxa and soil ecological functions. In addition, cross-kingdom SynComs may create opportunities for horizontal transfer of antibiotic-resistance or toxin-biosynthesis genes among introduced and resident microorganisms, although field-based evidence on the frequency, spatial extent, and ecological significance of such events remains limited.

Current technologies are also limited in their capacity for strain-specific tracing and long-term monitoring following field release, owing to the absence of low-cost, highly sensitive strain-specific labeling and quantification methods. As a result, it remains difficult to fully characterize the migration and persistence patterns of SynCom members across soil profiles, runoff, and crop residues, or to assess the risk of cross-boundary ecological dispersal mediated by irrigation and fertilizer movement. Moreover, no unified safety evaluation framework or regulatory standard has yet been established for SynCom products, and standardized testing protocols covering strain compatibility, gene-transfer potential, non-target toxicity, and long-term soil ecological legacy effects are lacking. Developing risk-classification systems, environmental-release procedures, and long-term ecological monitoring frameworks will be essential for the standardized and safe deployment of SynComs.

### 6.5 Toward precision SynCom application

In response to the challenges outlined above, SynCom research is undergoing a fundamental transition from traditional trial-and-error approaches toward a data-driven paradigm of rational design. This transition is underpinned by the deep integration of multi-omics technologies-genomics, transcriptomics, proteomics, and metabolomics-with computational modeling, aiming to achieve a holistic understanding of microbial interactions and community function. Combined with artificial intelligence and machine learning algorithms, this integrated approach enables the prediction of functional interactions under specific soil and crop conditions, thereby guiding the precise construction of SynComs.

Looking ahead, the development of SynCom technology should focus on establishing standardized community design principles, protocols, and benchmarking systems; deepening the integration of artificial intelligence with multi-omics technologies; strengthening the ecological adaptability and stability of SynComs; pursuing the directed design of SynComs tailored to specific agricultural challenges, such as continuous-cropping obstacles, soil salinization, and drought stress; and advancing scalable, low-cost industrial production technologies. Together, these approaches support a data-driven, iterative workflow in which omics-based candidate discovery, model-guided community assembly, greenhouse and field validation, and feedback-based redesign are integrated to improve the predictability and field robustness of SynComs (Figure 3).

**FIGURE 3:**
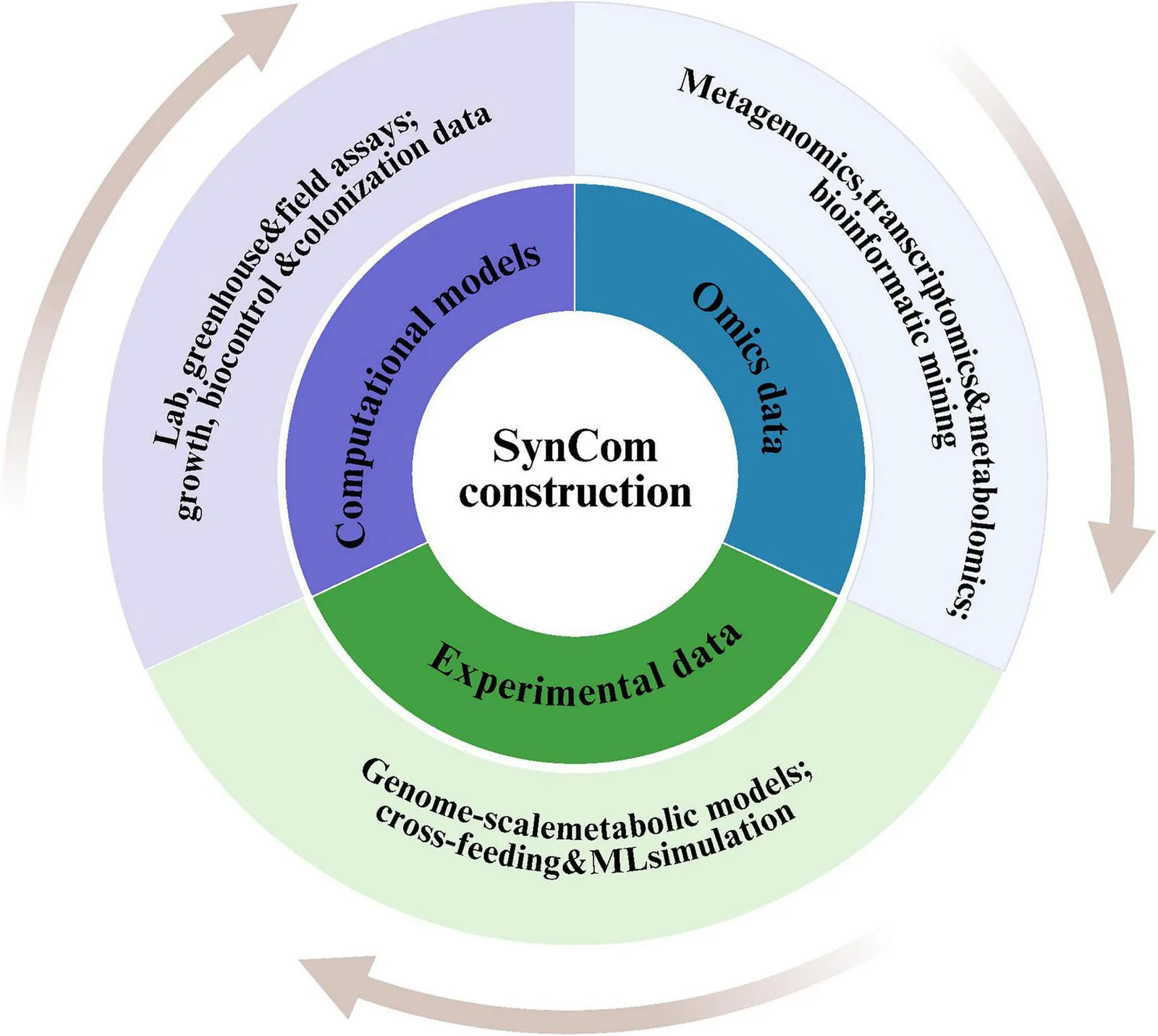
Data-driven iterative workflow for rational SynCom construction. SynCom construction is achieved through the iterative integration of omics data, experimental plant data, and computational models. Multi-omics analyses reveal microbial diversity, functional traits, and metabolic potential, whereas laboratory, greenhouse, and field assays validate plant growth-promoting, biocontrol, and colonization performance. Computational modeling, including genome-scale metabolic modeling, cross-feeding simulation, and machine-learning prediction, guides the selection and optimization of compatible microbial members. Feedback from experimental and omics data further refines model predictions, facilitating the construction of stable and functional SynComs. Created in BioRender. Cai, W. (2026), https://BioRender.com/bn04dso. BioRender Academic Publication License, Agreement No. 4HEU1EKJ2W.

## 7 Conclusions and future perspectives

SynComs are increasingly recognized as an effective strategy for plant growth promotion and soil-borne disease management owing to their functional complementarity, interspecific cooperation, and ecological synergy. However, current evidence does not indicate that SynComs are universally superior to single-strain inoculants. Their performance depends on community composition, ecological compatibility, inoculum design, and host–niche fit. No single construction strategy is likely to satisfy all requirements for colonization, complementarity, compatibility, controllability, and practical deployment, supporting the integration of ecological selection, interaction testing, and model-guided design.

Despite the promising performance of SynComs in laboratory experiments and small-scale field trials, their transition from experimental systems to large-scale agricultural application remains constrained by several major challenges. More broadly, the main challenge is no longer simply demonstrating biological potential under controlled conditions, but establishing reproducible performance across variable soils, crop genotypes, climates, and resident microbiomes. The lack of reliable predictive frameworks makes it difficult to quantitatively evaluate or forecast SynCom-mediated growth promotion and disease suppression across different soil types, crop genotypes, and environmental contexts. In addition, industrial-scale fermentation, formulation, and product development remain technically and economically challenging. In particular, immature large-scale fermentation processes, the high cost of microencapsulation and long-term storage, and the absence of standardized quality-control and regulatory frameworks continue to hinder the broad field deployment of SynCom-based products.

Among these challenges, multi-environment field validation should receive the highest priority. Replicated multi-location and multi-season trials are needed to determine whether reported benefits are reproducible under realistic agricultural conditions. At the same time, strain-resolved studies should clarify how inoculum dose, strain ratios, community composition, and temporal changes influence persistence and function. More standardized reporting, including appropriate controls, uncertainty estimates, comparisons with individual strains, and null or unsuccessful outcomes, would strengthen the evidence base and reduce publication and selection bias. Progress toward practical application will also depend on scalable fermentation, formulation, storage, and quality-control procedures, together with early integration of biosafety assessment, environmental monitoring, and regulatory considerations.

Future SynCom research should shift from descriptive community assembly toward predictive, testable, and field-validated design. Progress in plant SynComs will depend on the integration of ecological theory, strain-resolved experimentation, formulation science, field validation, and biosafety-by-design, which together will facilitate the transition from promising laboratory consortia to reliable, scalable, and safe microbiome-based agricultural technologies. Ultimately, rationally designed SynComs are expected to become an important component of sustainable agriculture by enhancing crop resilience, reducing agrochemical inputs, restoring soil health, and supporting the long-term ecological management of plant diseases. The long-term value of SynCom technology will therefore depend less on increasing community complexity than on developing communities whose establishment, function, safety, and manufacturability can be reliably demonstrated under realistic agricultural conditions.
